# Deletion of Insulin-Degrading Enzyme Elicits Antipodal, Age-Dependent Effects on Glucose and Insulin Tolerance

**DOI:** 10.1371/journal.pone.0020818

**Published:** 2011-06-09

**Authors:** Samer O. Abdul-Hay, Dongcheul Kang, Melinda McBride, Lilin Li, Ji Zhao, Malcolm A. Leissring

**Affiliations:** 1 Department of Neuroscience, Mayo Clinic Florida, Jacksonville, Florida, United States of America; 2 Department of Molecular Therapeutics, The Scripps Research Institute, Jupiter, Florida, United States of America; University of Padova, Italy

## Abstract

**Background:**

Insulin-degrading enzyme (IDE) is widely recognized as the principal protease responsible for the clearance and inactivation of insulin, but its role in glycemic control in vivo is poorly understood. We present here the first longitudinal characterization, to our knowledge, of glucose regulation in mice with pancellular deletion of the *IDE* gene (IDE-KO mice).

**Methodology:**

IDE-KO mice and wild-type (WT) littermates were characterized at 2, 4, and 6 months of age in terms of body weight, basal glucose and insulin levels, and insulin and glucose tolerance. Consistent with a functional role for IDE in insulin clearance, fasting serum insulin levels in IDE-KO mice were found to be ∼3-fold higher than those in wild-type (WT) controls at all ages examined. In agreement with previous observations, 6-mo-old IDE-KO mice exhibited a severe diabetic phenotype characterized by increased body weight and pronounced glucose and insulin intolerance. In marked contrast, 2-mo-old IDE-KO mice exhibited multiple signs of improved glycemic control, including lower fasting glucose levels, lower body mass, and modestly enhanced insulin and glucose tolerance relative to WT controls. Biochemically, the emergence of the diabetic phenotype in IDE-KO mice correlated with age-dependent reductions in insulin receptor (IR) levels in muscle, adipose, and liver tissue. Primary adipocytes harvested from 6-mo-old IDE-KO mice also showed functional impairments in insulin-stimulated glucose uptake.

**Conclusions:**

Our results indicate that the diabetic phenotype in IDE-KO mice is not a primary consequence of IDE deficiency, but is instead an emergent compensatory response to chronic hyperinsulinemia resulting from complete deletion of IDE in all tissues throughout life. Significantly, our findings provide new evidence to support the idea that partial and/or transient inhibition of IDE may constitute a valid approach to the treatment of diabetes.

## Introduction

Following its secretion from the pancreas, circulating insulin levels are regulated entirely by proteolytic degradation and other catabolic processes. The degradation and inactivation of insulin, in turn, is well established to be mediated primarily by insulin-degrading enzyme (IDE), a structurally and evolutionarily distinctive zinc-metalloprotease [1], [2], [3], [4]. Despite IDE's central involvement in insulin metabolism, surprisingly little work has directly addressed its role in regulating insulin and glucose homeostasis *in vivo*. Moreover, the few studies that have investigated this topic appear to support contradictory conclusions. On the one hand, IDE's established role in insulin catabolism predicts that IDE inhibition should increase the half-life of circulating insulin, an effect that could be beneficial for the treatment of diabetes. This idea is supported by a body of work [5] that emerged following the discovery of IDE in 1949 [6]. Most compellingly, Mirsky and colleagues demonstrated that a purified endogenous inhibitor of IDE (non-proteinaceous, but of unknown identity) could potentiate the hypoglycemic action of insulin *in vivo* [7]. On the other hand, recent work in two different animal models appears to support the contradictory idea that reductions in IDE activity can in fact *induce* a diabetic phenotype. First, a widely used rodent model of diabetes—the Goto-Kakizaki (GK) rat [8]—was shown to harbor missense mutations in the *IDE* gene that decrease the efficiency of insulin degradation [9], [10]. Subsequently, mice with homozygous deletion of the *IDE* gene (IDE-KO mice) were found to exhibit pronounced glucose intolerance as well as hyperinsulinemia [4]. The discrepancy between these animal modeling studies and earlier work suggesting a beneficial role for reducing IDE activity has not been resolved.

To address this topic, we characterized insulin and glucose homeostasis in IDE-KO and wild-type (WT) mice longitudinally, across the first 6 months of life. Confirming earlier work [4], by 6 months of age IDE-KO mice develop pronounced fasting hyperinsulinemia and glucose intolerance and, as we now show, significant insulin intolerance, fasting hyperglycemia and increased body mass. In striking contrast, at 2 months of age IDE-KO mice were significantly improved relative to WT controls in terms of virtually all parameters tested, including body mass, fasting blood glucose, as well as glucose and insulin tolerance, while showing the same degree of hyperinsulinemia as 6-mo-old IDE-KO mice. Consistent with other models of chronic hyperinsulinemia, IDE-KO mice exhibited reductions in insulin receptor (IR) levels and/or function in multiple tissues that, like the diabetic phenotype itself, emerged in an age-dependent manner. These results suggest that the diabetic phenotype in IDE-KO mice emerges as a compensatory adaptation to chronic hyperinsulinemia induced by complete, pan-cellular deletion of IDE throughout life. The implications of these findings for the pathogenesis of type 2 diabetes and for the therapeutic potential of IDE inhibition are discussed.

## Results

To elucidate the consequences of IDE deficiency on glycemic regulation *in vivo*, we investigated insulin and glucose homeostasis in male IDE-KO mice and WT littermates backcrossed into a pure C57Bl/6J genetic background. We used a longitudinal design, in which a single group of animals was monitored repeatedly at 3 successive ages (2, 4 and 6 months) in terms of multiple diabetes-relevant parameters. For biochemical analyses, a separate set of mice was harvested cross-sectionally, at 2 and 6 months of age.

**Figure 1:**
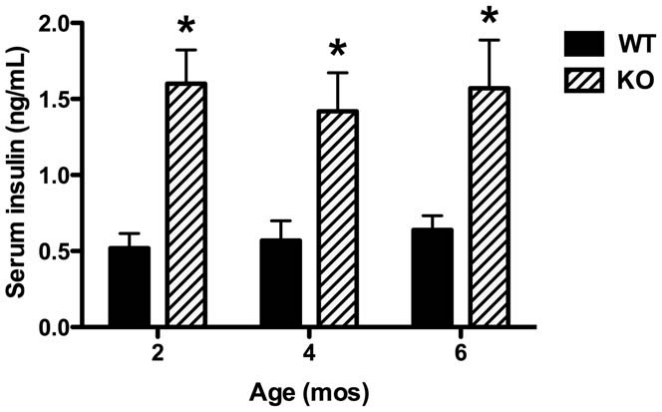
Constitutive hyperinsulinemia in IDE-KO mice. Insulin levels in serum of 2-, 4- and 6-mo-old wild-type (WT) and IDE-KO (KO) mice following overnight fasting. Data are mean ± SEM of 10–12 mice per group. *P<0.05 determined by 2-tailed Student's t test.

**Figure 2:**
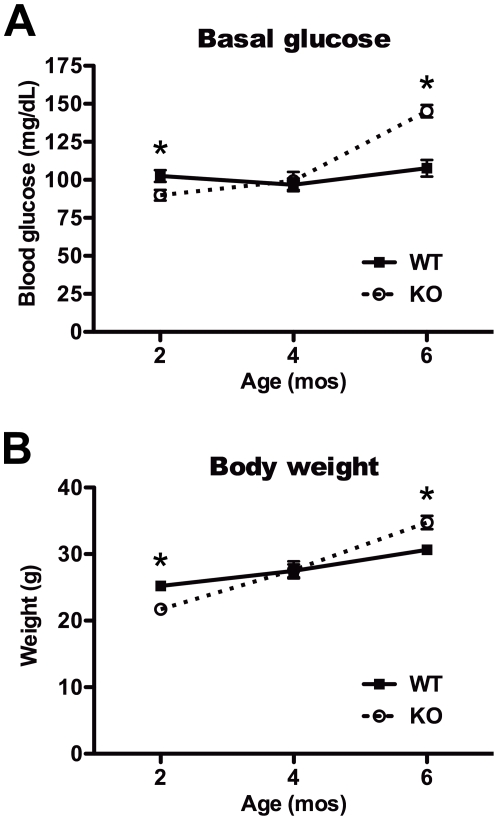
Age-dependent changes in basal blood glucose and body weight. ***A***, Blood glucose in 2-, 4- and 6-mo-old wild-type (WT) and IDE-KO (KO) mice following overnight fasting. Note that 2-mo-old IDE-KO mice exhibit significantly lower basal glucose levels relative to controls. ***B***, Body weight of fasted 2-, 4- and 6-mo-old WT and IDE-KO mice. Note that IDE-KO mice weigh significantly less than wild-type controls mice at 2 months yet significantly more at 6 months of age. Data are mean ± SEM of 10–12 mice per group. *P<0.05 IDE-KO *vs.* WT as determined by 2-tailed Student's t test.

As expected given the known role of IDE in insulin catabolism, and consistent with previous findings [4], fasting serum insulin levels in IDE-KO mice were found to be approximately 3-fold higher than those in WT controls at all ages examined (Fig. 1). By contrast, the effects of IDE deficiency on virtually all other parameters varied in an age-dependent manner. Relative to age-matched controls, basal glucose levels were significantly lower in 2-mo-old IDE-KO mice, unchanged in 4-mo-old animals, and significantly increased in 6-mo-old IDE-KO mice (Fig. 2A). Body mass followed a corresponding trend, with 2-mo-old IDE-KO mice being significantly lighter, and 6-mo-old IDE-KO mice significantly heavier, than age-matched WT controls (Fig. 2B).

**Figure 3:**
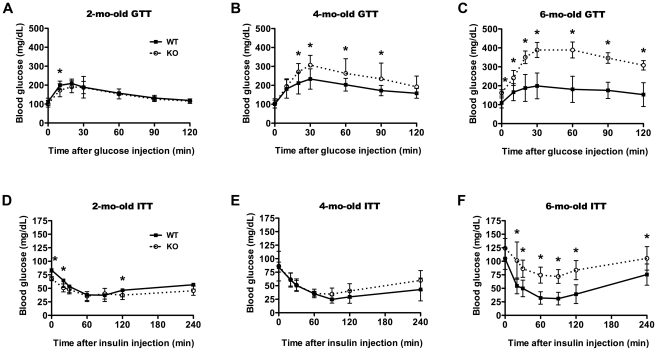
Age-dependent emergence of the diabetic phenotype in IDE-KO mice. ***A***–***C***, Glucose tolerance tests in 2- (***A***), 4-(***B***), and 6-mo-old (***C***) wild-type (WT) and IDE-KO (KO) mice. Note that 2-mo-old IDE-KO mice exhibit improved glucose tolerance at some time points. Data are mean ± s.d. of 10–12 mice per condition. *P<0.05. ***D***–***F***, Insulin tolerance tests in 2- (***D***), 4-(***E***), and 6-mo-old (***F***) WT (WT) and IDE-KO (KO) mice. Note the trend towards improved insulin tolerance in 2-mo-old IDE-KO mice. Data are mean ± s.d. of 10–12 mice per condition. *P<0.05 IDE-KO *vs.* WT as determined by 2-tailed Student's t test.

The emergence of the diabetic phenotype induced by IDE deletion [4] was monitored by performing glucose and insulin tolerance tests at 2, 4 and 6 months of age. Corroborating previous results [4], at 6 months of age IDE-KO mice exhibited profound glucose intolerance, with mean blood glucose levels induced by intraperitoneal injection of D-glucose (1 g/kg) approaching 400 mg/dL by 30 min as compared to 200 mg/dL for WT mice (Fig. 3C). By contrast, at 2 months of age IDE-KO mice not only lacked glucose intolerance but in fact exhibited modest but statistically significant improvements in glucose disposal (Fig. 3A). Consistent with an age-dependent transition from improved to impaired glucose tolerance, 4-mo-old IDE-KO mice showed an intermediate phenotype (Fig. 3B). In terms of insulin tolerance, the IDE-KO mice were significantly intolerant at 6 months (Fig. 3F), but largely normal at 4 months of age relative to age-matched controls (Fig. 3E). Notably, 2-mo-old IDE-KO mice showed statistically significant improvements in insulin tolerance relative to WT littermate controls (Fig. 3D).

**Figure 4:**
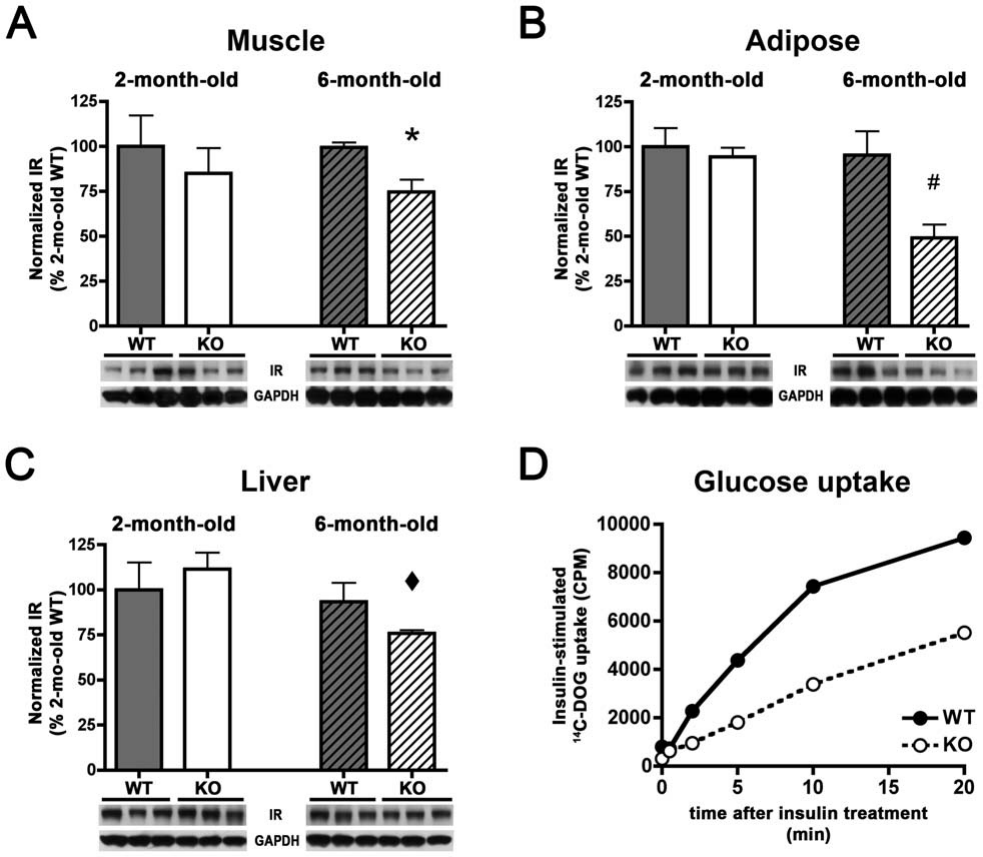
IDE-KO mice show age-dependent reductions in IR levels and insulin-stimulated glucose uptake that correlate with the onset of the diabetic phenotype. ***A***–***C***, IR levels in muscle (***A***) adipose (***B***) and liver (***C***) tissue from wild-type (WT) and IDE-KO (KO) mice. Note that IR levels in IDE-KO mice are significantly decreased in all tissues at 6 months, but not at 2 months of age. Graphs show mean ± SEM of IR levels quantified by luminescent imaging (see *Materials and Methods*) and normalized to IR levels in 2-mo-old WT mice. *P<0.05 relative to 6-mo-old WT mice; ^#^P<0.01 relative to 2-mo-old KO mice and P<0.05 relative to 6-mo-old WT and KO mice; ^⧫^P<0.05 relative to 2-mo-old KO mice, all determined by 2-tailed Student's t tests. ***D***, Insulin-stimulated glucose uptake is significantly impaired in primary adipocytes isolated from 6-mo-old IDE-KO mice. Quantification was performed by measuring cellular uptake of ^14^C-deoxyglucose (^14^C-DOG) various times after addition of insulin (16.7 nM). Data are mean of 2 independent experiments utilizing epididymal adipose tissue collected from 4 mice per condition for each replication.

The preceding results suggest that the diabetic phenotype in older IDE-KO mice may represent a compensatory response to chronically elevated circulating insulin. To explore this idea at a biochemical level, tissues were harvested from a separate set of mice at 2 and 6 months of age. Based on evidence from other animal models of hyperinsulinemia [11], [12], [13] (see below), we focused on insulin receptor (IR) levels, which were quantified in multiple tissues involved in the utilization [14] and clearance [15] of insulin. At 2 months of age, WT and IDE-KO mice exhibited comparable IR levels in all tissues examined (Fig. 4A–C). IR levels also did not change significantly between 2 and 6 months of age in any tissues from WT mice (Fig. 4A–C). In marked contrast, 6-mo-old IDE-KO mice showed significant reductions in total IR levels in all tissues examined, including muscle (Fig. 4A), adipose tissue (Fig. 4B) and liver (Fig. 4C).

To determine whether these biochemical perturbations were associated with functional changes to glucose transport, we conducted glucose uptake assays in primary adipocytes isolated from 6-mo-old IDE-KO and WT mice. Consistent with the reduction in IR levels observed by western blotting, IDE-KO adipocytes showed substantial impairments in insulin-stimulated glucose uptake (Fig. 4D).

## Discussion

In the present study, we investigated glucose homeostasis in mice lacking IDE, the principal protease involved in the clearance and inactivation of insulin [15], across the first 6 months of life. Consistent with its known functional role, IDE deficiency led to significant hyperinsulinemia at all ages examined (2, 4, and 6 months). However, the elimination of IDE-mediated insulin catabolism had remarkably different consequences for glucose homeostasis at different ages. In the youngest animals studied, IDE deficiency led to modestly improved glycemic control and decreased body weight, a phenotype that is consistent with earlier experimental findings showing that IDE inhibition enhances the hypoglycemic action of insulin *in vivo* [7], [16]. On the other hand, as the IDE-KO animals aged, they showed progressively poorer glycemic control, culminating in profound glucose and insulin intolerance. These metabolic disturbances correlated with age-dependent decreases in IR levels in multiple tissues, together with functional impairments in IR-mediated glucose uptake.

Taken together, these findings have significant implications for both the etiology and the potential treatment of diabetes. In terms of diabetes pathogenesis, our results corroborate several studies suggesting that insulin resistance can arise as a secondary consequence of primary persistent hyperinsulinemia. For instance, repeated administration of insulin (up to 6U/day) to rats over a 2-week period led to reductions in both maximal insulin binding capacity and IR-mediated glucose utilization [11]. Moreover, a 4-week period of hyperinsulinemia induced by surgical diversion of pancreatic veins from the portal vein to the vena cava in dogs resulted in significant reductions in the glucose disposal rate that were associated with reduced IR activity [12]. Furthermore, hyperinsulinemia induced by transgenic overexpression of human insulin has been reported to result in impaired glucose tolerance [13]. Taken together with our own findings in IDE-KO mice, this body of work strongly suggests that, if sufficient in magnitude and duration, chronic hyperinsulinemia *per se* can be a primary cause of insulin resistance. Mechanistically, in light of the finding that IR levels and/or activity are significantly reduced in IDE-KO mice and multiple other models of chronic hyperinsulinemia, we speculate that insulin insensitivity may, in part, result from classic receptor down-regulation—*i.e.*, adaptation to chronically elevated insulin levels.

While the disturbances in IR levels and functionality we observed appear to offer a neat explanation for the emergence of the diabetic phenotype in IDE-KO mice, we emphasize that considerably more study will be required before firm conclusions can be made. For example, until a more comprehensive analysis is conducted, we cannot formally exclude the possibility that deletion of IDE could trigger defects in insulin signaling components that in turn elicit compensatory hyperinsulinemia. Given that IDE is known to degrade other substrates involved in glucose homeostasis and diabetes pathogenesis, including glucagon [17] and amylin [18], this question merits further investigation.

The reduction in liver IR levels seen in IDE-KO mice are of special interest, because clearance of circulating insulin, which occurs primarily in liver, is initiated and rate-limited by IR-mediated internalization [3]. Given this functional role, reductions in liver IR levels would be predicted to result in impaired insulin clearance and increased basal insulin levels. Because both of these disturbances are hallmark features of type 2 diabetes, liver IR levels may represent a critical factor in the etiology and potential treatment of these aspects of the disease. It is relevant to note in this context that, in immortalized IDE-KO hepatocytes maintained in culture for multiple passages, IR levels were found to be dramatically *increased* (∼8-fold) relative to WT hepatocytes, with the population of cell-surface IR elevated still further (∼40-fold; J.Z. and M.A.L., unpublished observations). While a comparable effect was not observed *in vivo*, this observation implies that hepatic IR levels are indeed regulated by IDE-dependent factors that, if identified, could potentially be exploited to modulate insulin clearance.

The precise mechanisms underlying the enhancement of insulin signaling induced by transient reductions in IDE are presently unclear. Superficially, this effect might appear to exclusively derive from the slowing of insulin clearance from the circulation (see Ref. 5). However, recent evidence suggests that suppression of IDE activity can also enhance insulin signaling subsequent to the binding of insulin to the IR [19], suggesting that processes occurring at the level of destination cells may be operative either alternatively or in addition to effects on circulating insulin. More work will be needed to clarify the relative contributions of these two possible mechanisms.

From a therapeutic perspective, our results support the idea that pharmacologic inhibition of IDE, properly implemented, may represent a viable—even attractive—therapeutic approach to the treatment of diabetes. In terms of effects on insulin signaling, a short-acting, partial inhibitor of IDE would be predicted to exert similar effects as sulfonylureas or similar secretagogues—namely, increasing the area under the curve of circulating insulin, while maintaining normal rhythmicity of secretion. It is fascinating to note in this context that the sulfonylureas were originally believed to act by inhibiting IDE [20]. To the extent that IDE inhibitors and secretagogues both act by increasing circulating insulin, the mechanism-based risk-benefit profile of both would likely be similar. However, IDE inhibitors might hold certain advantages over secretagogues, for they would not be predicted to affect pancreatic function *per se*. Moreover, the risks from overdose of an IDE inhibitor would appear to be minimal, since complete inhibition of IDE does not have immediate deleterious consequences [4]. This compares favorably with many other anti-diabetic medications that can be harmful or even lethal if taken in excess [21]. It is relevant to note in this context that our laboratory recently succeeded in developing peptide hydroxamate IDE inhibitors, which represent the first potent and selective small-molecule inhibitors of IDE or indeed any other member of this unusual protease superfamily [19]. We are actively working to generate improved versions of these first-generation IDE inhibitors sufficiently stable for use *in vivo*, which will permit a direct test of the anti-diabetic potential of this important new class of protease inhibitor.

In conclusion, our analysis of IDE-KO mice suggests a natural resolution to the conflicting ideas concerning the consequences of IDE inhibition for glucose homeostasis *in vivo*: by virtue of IDE's role in insulin catabolism, short-term and/or partial inhibition of IDE is expected to enhance the hypoglycemic action of insulin, an effect that in principle could be beneficial for the treatment of diabetes. Conversely, sustained, complete inhibition of IDE in all tissues, by inducing chronic hyperinsulinemia, elicits compensatory changes to insulin signaling that contribute to insulin intolerance and, hence, glucose intolerance. Given the beneficial effects of transient inhibition of IDE on glycemic control observed in this study, pharmacological inhibition of IDE would appear to represent a potentially viable approach to the treatment of diabetes that merits more thorough evaluation in the future.

## Materials and Methods

### Animals

Founder IDE^+/−^ mice were obtained from the Mutant Mouse Regional Resource Center then backcrossed into the C57Bl6/J background for 9 generations prior to initiation of the study. Mice were fed Teklad S-2335 mouse breeder chow (Harlan Laboratories, Indianapolis, IN) and males were used for all experiments. This study was carried out in strict accordance with the recommendations in the Guide for the Care and Use of Laboratory Animals of the National Institutes of Health. The protocol was approved by the Institutional Animal Care and Use Committee of Mayo Clinic Jacksonville (Protocol Number A16810). All efforts were made to minimize suffering.

### Insulin and glucose measurements

Serum insulin levels were determined using the HTRF® Insulin Assay (CisBio, Bedford, MA) and detected using a SpectraMax M5e microplate reader (Molecular Devices, Inc., Sunnyvale, CA) according to manufacturers' recommendations. Blood glucose levels were measured using the Ascencia® ELITE® SL Blood Glucose Monitoring System (Bayer HealthCare, Tarrytown, NY) according to manufacturer's recommendations. Glucose and insulin tolerance tests were conducted by monitoring blood glucose following i.p. administration of D-glucose (1 g/kg) or human insulin (1 U/kg), respectively. All measurements were obtained from tail blood samples of mice fasted for 6 to 9 h.

### Western blotting

Western blots were performed as described [22] using protein extracts (8 µg/well) from muscle, adipose and liver tissue. Antibodies and dilutions used included anti-Insulin Receptor (1∶1000; Cell Signaling Technology, Danvers, MA), and anti-GAPDH (1∶10,000; BioDesign, Inc., Carmel, NY). Western blots of IR and loading controls were quantified using a LAS-4000 Luminescent Image Analyzer equipped with a 3.2-megapixel CCD camera and its associated software (MultiGauge v. 3.1) according to manufacturer's recommendations (Fujifilm Lifescience, Greenwood, SC).

### Glucose uptake assays

Glucose uptake assays were performed in primary adipocytes isolated from epididymal fat pads essentially as described [23]. Briefly, dissociated adipocytes were incubated in KRBH buffer (120 mM NaCl, 4 mM KH_2_PO_4_, 2 mM CaCl_2_, 1 mM MgSO_4_, 30 mM HEPES, pH 7.4) supplemented with adenosine (200 nM). Glucose uptake was assessed by addition of KRBH buffer supplemented with 2.5 mM 2-D-glucose, 1% BSA, and 1 µM ^14^C-deoxy-D-glucose (^14^C-DOG; Perkin Elmer, Waltham, MA) following stimulation with human recombinant insulin (16.7 nM). Reactions were incubated at 37°C and terminated by addition of cytochalasin B. Following partitioning of cellular contents in dinoyl phthalate, radioactivity in the non-aqueous layer was counted using a liquid scintillation counter (Beckman Coulter).
